# The association between weekend catch-up sleep and obesity in U.S. adults: A cross-sectional analysis of NHANES 2017–2020

**DOI:** 10.1097/MD.0000000000044670

**Published:** 2025-09-19

**Authors:** Jinfang Zeng, Dongxiao Huang, Minmin Zhu, Jinjin Jian

**Affiliations:** a Department of Anesthesiology and Pain Medicine, Wuxi No. 2 People’s Hospital, Wuxi, China; b Department of Anesthesiology, Affiliated Hospital of Jiangnan University, Wuxi, China.

**Keywords:** circadian rhythm, metabolic association, NHANES, obesity, sleep duration, sleep irregularity, weekend catch-up sleep

## Abstract

Weekend catch-up sleep (WCS) is a behavioral strategy employed to compensate for weekday sleep loss and has been reported to be associated with various metabolic outcomes. However, its association with obesity remains controversial. This study aimed to investigate the relationship between WCS and obesity among U.S. adults using data from the National Health and Nutrition Examination Survey. We analyzed data from 8279 individuals aged 20 years and older, derived from the 2017–2020 cycles of the National Health and Nutrition Examination Survey database. WCS was defined as the difference between self-reported sleep duration on weekends and weekdays, and subsequently divided into quartiles. Obesity was identified based on a body mass index threshold of 30 kg/m² or higher. To examine the relationship between WCS and obesity, we employed survey-weighted logistic regression models, treating WCS as a continuous variable. Restricted cubic spline curves and two-piecewise regression models were additionally explored for sensitivity, but statistical tests did not support a nonlinear association. Stratified analyses and interaction tests were performed as exploratory assessments to evaluate potential effect modifiers. Each additional hour of WCS was associated with a modestly higher odds of obesity (odds ratio = 1.03, 95% confidence interval: 1.00–1.06; *P* = .028) after adjusting for demographic, socioeconomic, behavioral, and clinical factors. Statistical tests did not support a nonlinear relationship (*P* for nonlinearity = .816), indicating that the association is best characterized as linear. Exploratory subgroup analyses suggested potentially stronger associations in females, non-Hispanic Black individuals, and nonsmokers; however, these findings were not statistically robust and should be interpreted with caution. WCS was positively associated with obesity in U.S. adults in a modest linear manner. Expl oratory models suggested a possible threshold, but statistical tests (*P* for nonlinearity = .816; likelihood ratio test *P* = .113) did not support this. Thus, the linear model provides the most parsimonious description of the data. These findings suggest that while moderate WCS may reflect an attempt to compensate for sleep debt, excessive or irregular sleep extension was associated with higher odds of obesity. However, given the cross-sectional design, these results indicate association rather than causation.

## 1. Introduction

Obesity is a complex and long-term health condition characterized by abnormal or excessive fat accumulation. It is commonly defined by a body mass index (BMI) equal to or exceeding 30 kg/m². The prevalence of obesity has increased globally to epidemic levels.^[1]^ As reported by the World Health Organization, over 650 million adults are currently living with obesity, representing more than 13% of the global adult population.^[2]^ In the United States, nearly 4 in 10 adults are affected, highlighting a major public health concern despite ongoing prevention efforts.^[3]^

Obesity has been associated with higher mortality and with numerous health conditions, such as type 2 diabetes, cardiovascular conditions, hypertension, sleep-disordered breathing like obstructive sleep apnea (OSA), several forms of cancer, and psychological disorders.^[4]^ In addition, obesity is linked to substantial economic burdens, including increased healthcare utilization, productivity loss, and disability.^[5]^ Although lifestyle modification, pharmacotherapy, and bariatric surgery are available treatment options, their long-term effectiveness is often limited due to poor adherence and variability in individual responses.^[6]^ These challenges underscore the importance of research to better understand factors associated with obesity.

Sleep has been increasingly studied as a potentially important factor associated with obesity and metabolic regulation. A growing body of evidence has reported associations between insufficient sleep duration and obesity as well as related metabolic disorders.^[7]^ However, many studies focus solely on average sleep duration, failing to account for weekly variations in sleep patterns. Traditional sleep metrics, such as mean or weekday-only sleep duration, may not adequately reflect real-life sleep behavior, potentially leading to exposure misclassification and biased associations.^[8]^

In this context, WCS – the additional sleep obtained on nonworking days relative to weekdays – has gained attention as a behaviorally relevant indicator of sleep compensation. Prior studies have suggested that WCS might be associated with metabolic processes such as insulin sensitivity, appetite regulation, and inflammation.^[9,10]^ Compared to static average sleep metrics, WCS provides a more dynamic marker for evaluating sleep-related health factors in population-based research.

Nevertheless, the long-term effects of WCS on obesity remain unclear. While some studies have reported potentially favorable metabolic associations of moderate WCS,^[9,11]^ others have suggested possible adverse outcomes related to circadian disruption.^[12,13]^ These inconsistencies highlight the need for further investigation using large, representative datasets.

To address this gap, this study systematically examined the association between WCS and obesity using a nationally representative National Health and Nutrition Examination Survey (NHANES) population sample. By adjusting for multiple covariates and conducting subgroup and threshold analyses, we aimed to clarify the relationship between WCS and obesity, including potential nonlinear patterns. These findings provide epidemiological evidence and may inform future research on the role of sleep patterns in relation to obesity.

## 2. Materials and methods

### 2.1. Study population

The present analysis draws on data from NHANES, a cross-sectional health survey aimed at providing nationally representative estimates for the U.S. civilian, noninstitutionalized population. Overseen by the National Center for Health Statistics (NCHS) under the Centers for Disease Control and Prevention, NHANES employs a complex, multistage sampling strategy to ensure population-level validity. Data are gathered through household interviews, complemented by detailed physical examinations and laboratory analyses conducted in mobile health units.^[14]^ For the current analysis, we included data from 2017 to 2020, which contained detailed information on self-reported sleep duration, anthropometric measurements, and relevant demographic and lifestyle variables.

**Figure 1.**
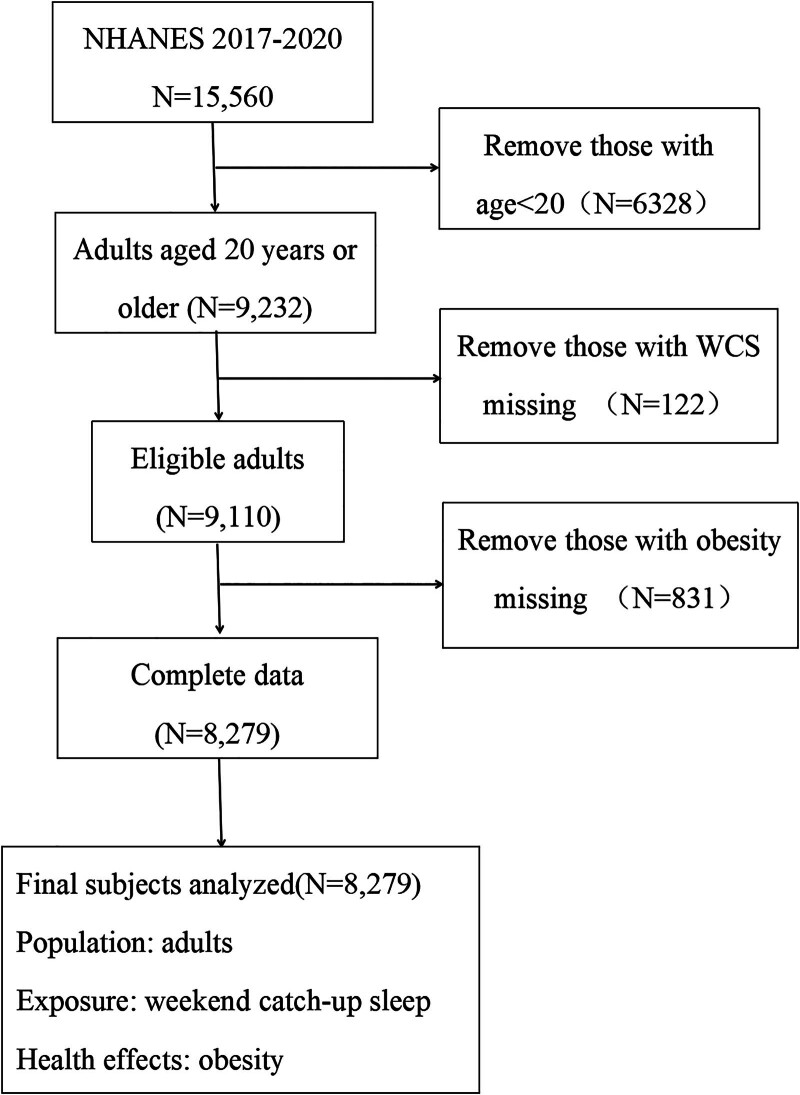
Flow diagram of the inclusion criteria and exclusion criteria. NHANES = National Health and Nutrition Examination Survey, WCS = weekend catch-up sleep.

For this study, we used data from the NHANES 2017–2020 cycles, which initially included 15,560 participants. We excluded individuals under 20 years of age (n = 6328), those with missing information on weekend catch-up sleep (WCS; n = 122), and those without obesity data (n = 831). After applying these exclusion criteria, a total of 8279 adults with complete data on exposure, outcome, and covariates were included in the final analysis (Fig. 1). For covariates with missing values, categorical variables were recoded based on the distribution of observed responses, while continuous variables were imputed using mean substitution. This approach was applied to preserve the sample size and minimize potential bias introduced by missing covariate data. The NHANES study protocols received ethical approval from NCHS. All individuals who participated in the survey provided written informed consent prior to data collection. To protect participant confidentiality, all data are fully anonymized and can be accessed freely through the official website of the Centers for Disease Control and Prevention: https://www.cdc.gov/nchs/nhanes/.^[15]^

### 2.2. Obesity

Obesity status was assessed using the BMI, derived by dividing an individual’s weight (in kilograms) by the square of their height (in meters). These measurements were obtained through standardized anthropometric procedures conducted as part of the NHANES physical examination protocol. Trained health professionals measured height and weight following uniform national protocols to ensure consistency and reliability. According to the World Health Organization classification, participants with a BMI of 30.0 kg/m² or higher were classified as obese.^[16]^

### 2.3. Measurements of WCS

WCS was derived from participants’ self-reported sleep habits obtained through the NHANES sleep questionnaire (2017–2020 cycles). Individuals provided typical bedtimes and wake-up times separately for weekdays (Monday–Friday) and weekends (Saturday–Sunday), from which average nightly sleep durations were calculated. WCS was defined as the difference between mean weekend and weekday sleep durations (weekend minus weekday), consistent with previously established methods.^[17]^ A positive WCS value indicated additional sleep on weekends (i.e., sleep compensation), whereas a negative value reflected a weekend sleep deficit. Implausible values, such as sleep durations exceeding 16 hours per night, were excluded. WCS was modeled as a continuous variable in all regression analyses, to preserve the full range of variation and avoid information loss that may result from arbitrary categorization.^[18]^

### 2.4. Covariates

The selection of covariates was informed by prior literature and their potentially confounding effects on the association between WCS and obesity. Demographic variables included age as a continuous variable, biological sex classified as male or female, and race or ethnicity categorized into 5 groups: Non-Hispanic White, Non-Hispanic Black, Mexican American, Other Hispanic, and individuals of other or mixed racial backgrounds.^[19]^ Socioeconomic factors encompassed the highest level of educational attainment, grouped as less than high school, completion of high school or equivalent, and more than high school. Marital status was classified into 3 categories: currently married or living with a partner, formerly married including widowed, divorced, or separated, and never married. Economic status was assessed using the family poverty-income ratio (PIR), which was dichotomized according to whether it was below or above 200% of the federal poverty threshold.^[20]^ Behavioral characteristics included smoking status, defined as either smoking or nonsmoking, physical activity (assessed using NHANES physical activity questionnaires and categorized according to recommended guidelines), and total energy intake. To capture overall diet quality beyond total energy intake, the Healthy Eating Index (HEI) was used, derived from 24-hour dietary recall interviews conducted using standardized protocols developed by NHANES, based on the USDA’s Automated Multiple-Pass Method. Clinical variables included the presence of hypertension and diabetes(based on self-reported physician diagnoses or medication use), as well as OSA and depression status, both identified through self-report and standardized NHANES instruments.

Occupational factors included shift work status, categorized as day shift versus nonstandard shifts (evening, night, rotating, or other), given its established association with circadian misalignment and obesity risk.

All covariates were defined and measured following NHANES guidelines to ensure methodological consistency and data reliability. Before model fitting, multicollinearity among covariates was assessed using the variance inflation factor. A variance inflation factor value >10 was considered indicative of serious multicollinearity. No covariates exceeded this threshold, suggesting that multicollinearity was not a concern in the final regression models.

### 2.5. Statistical analyses

All analyses were conducted using R (version 4.3.0), Zstats v1.0 (www.zstats.net), and EmpowerStats (version 5.0). To account for the complex, multistage sampling design of NHANES, all procedures incorporated the appropriate 4-year sampling weights, strata, and primary sampling units. This approach ensured nationally representative estimates by correcting for unequal selection probabilities, nonresponse, and post-stratification adjustments. Descriptive statistics were expressed as weighted means with standard errors for continuous variables and weighted proportions for categorical variables. The association between WCS and obesity was examined using survey-weighted logistic regression models, with odds ratios (ORs) and 95% confidence intervals (CIs) reported. Two multivariable models were constructed to control for confounding. Model 1 adjusted for age, sex, and race/ethnicity. Model 2 included additional adjustments for educational attainment, marital status, family PIR, hypertension, diabetes, smoking status, and total energy intake, physical activity, OSA, depression, shift work, HEI, encompassing key socioeconomic, behavioral, and clinical factors. To explore potential nonlinear relationships, we applied generalized additive models with penalized spline functions and fitted exploratory two-piecewise linear regression models. Inflection points were estimated using a recursive algorithm. However, formal model comparison (log-likelihood ratio test) showed that these more complex models did not significantly improve fit compared with a simple linear model. Therefore, the primary analysis and interpretation focus on the linear association. Subgroup analyses were conducted to assess potential effect modification across strata defined by sex, race/ethnicity, education level, marital status, smoking status, hypertension, diabetes, weekday sleep duration, and the presence or absence of WCS. Interaction terms were tested using likelihood ratio tests. Sensitivity analyses were performed to assess the robustness of the findings. First, the association between WCS and obesity was reevaluated using a complete-case analysis excluding all participants with missing covariate data. Second, alternative categorizations of WCS were applied to examine consistency across different exposure definitions. These analyses yielded results that were directionally consistent with the primary models. All statistical tests were two-sided, and a *P*-value < .05 was considered statistically significant.

No correction for multiple comparisons was applied in the subgroup analyses, which were strictly exploratory in nature. Given the increased risk of false-positive results under multiple testing, all subgroup findings should be interpreted with extreme caution and considered hypothesis-generating only, not confirmatory.

## 3. Results

### 3.1. Characteristics of the participants

A total of 8279 adults were included in the final analysis, comprising 3531 individuals with obesity and 4748 without obesity. Table 1 presents the weighted baseline characteristics stratified by obesity status. The overall mean age was 50.75 ± 17.49 years, with no significant age difference between the obese and nonobese groups (50.68 ± 16.56 vs 50.81 ± 18.14 years; *P* = .778). Total energy intake was similar across groups (2114.81 ± 958.30 vs 2139.51 ± 984.27 kcal/day; *P* = .366). Participants with obesity reported slightly longer WCS duration (0.73 ± 1.68 vs 0.64 ± 1.58 hours; *P* = .025), although no difference was observed in total weekend sleep time (*P* = .866). Gender distribution differed significantly (*P* < .001): a higher proportion of females was present in the obesity group (55.45% vs 48.86%), whereas males were more prevalent in the nonobese group (51.14% vs 44.55%). Significant racial disparities were noted (*P* < .001). Non-Hispanic Black individuals had a higher proportion in the obesity group (31.89% vs 22.60%), while those identifying as Other Race – including multi-racial – had a higher proportion in the nonobese group (22.45% vs 9.74%). Socioeconomic status also showed variation, with a greater proportion of participants in the obesity group having a PIR below 2 (41.04% vs 38.29%; *P* = .011). There were no significant differences in educational attainment (*P* = .177) or marital status (*P* = .102) between groups. Smoking prevalence did not differ significantly either (*P* = .168). Clinically, the prevalence of diabetes (24.19% vs 13.71%; *P* < .001) and hypertension (47.89% vs 31.09%; *P* < .001) was higher in the obesity group. In terms of weekday sleep patterns, participants with obesity more often reported < 6 hours of sleep per night (11.75% vs 10.32%; *P* = .039). No significant difference was observed in the distribution of negative vs nonnegative WCS (*P* = .399).

**Table 1:** Weighted baseline characteristics of patients obesity.

Variable	Total	Without obesity	With obesity	*P* value
N	8279	4748	3531	
Age (yrs)	50.75 ± 17.49	50.81 ± 18.14	50.68 ± 16.56	.778
Total energy intake (kcal)	2033.57 ± 761.38	2052.56 ± 767.44	2008.04 ± 752.51	.012
Healthy Eating Index	55.58 ± 12.34	58.47 ± 12.08	54.38 ± 12.67	<.001
Weekend sleep hours (h)	8.24 ± 1.79	8.25 ± 1.76	8.23 ± 1.83	.866
Weekend catch-up sleep (h)	0.68 ± 1.62	0.64 ± 1.58	0.73 ± 1.68	.025
Gender, n (%)				<.001
Male	4001 (48.33%)	2428 (51.14%)	1573 (44.55%)	
Female	4278 (51.67%)	2320 (48.86%)	1958 (55.45%)	
Race, n (%)				<.001
Mexican American	963 (11.63%)	483 (10.17%)	480 (13.59%)	
Other Hispanic	849 (10.25%)	490 (10.32%)	359 (10.17%)	
Non-Hispanic White	2858 (34.52%)	1636 (34.46%)	1222 (34.61%)	
Non-Hispanic Black	2199 (26.56%)	1073 (22.60%)	1126 (31.89%)	
Other Race – including Multi-Racial	1410 (17.03%)	1066 (22.45%)	344 (9.74%)	
Education level, n (%)				.177
Less than high school	1536 (18.55%)	897 (18.89%)	639 (18.10%)	
High school grad/GED or equivalent	1989 (24.02%)	1106 (23.29%)	883 (25.01%)	
Some college or AA degree	4754 (57.42%)	2745 (57.81%)	2009 (56.90%)	
Marital status, n (%)				.102
Married/Living with Partner	4831 (58.35%)	2815 (59.29%)	2016 (57.09%)	
Widowed/Divorced/Separated	1844 (22.27%)	1023 (21.55%)	821 (23.25%)	
Never married	1604 (19.37%)	910 (19.17%)	694 (19.65%)	
PIR				.011
<2	3267 (39.46%)	1818 (38.29%)	1449 (41.04%)	
≥2	5012 (60.54%)	2930 (61.71%)	2082 (58.96%)	
Hypertension, n (%)				<.001
Yes	3179 (38.40%)	1485 (31.28%)	1694 (47.98%)	
No	5100 (61.60%)	3263 (68.72%)	1837 (52.02%)	
Diabetes, n (%)				<.001
Yes	1508 (18.21%)	653 (13.75%)	855 (24.21%)	
No	6771 (81.79%)	4095 (86.25%)	2676 (75.79%)	
Depression				<.001
No	7586 (91.63%)	4433 (93.37%)	3153 (89.29%)	
Yes	693 (8.37%)	315 (6.63%)	378 (10.71%)	
Workdays sleep hours (h)				.039
<6	905 (10.93%)	490 (10.32%)	415 (11.75%)	
≥6	7374 (89.07%)	4258 (89.68%)	3116 (88.25%)	
Physical activity				<.001
Moderate	3626 (43.80%)	2000 (42.12%)	1626 (46.05%)	
Less	4653 (56.20%)	2748 (57.88%)	1905 (53.95%)	
Shift work				.709
No	5334 (64.43%)	3051 (64.26%)	2283 (64.66%)	
Yes	2945 (35.57%)	1697 (35.74%)	1248 (35.34%)	
OSA				<.001
No	4135 (49.95%)	2749 (57.90%)	1386 (39.25%)	
Yes	4144 (50.05%)	1999 (42.10%)	2145 (60.75%)	
Smoking, n (%)				.164
Yes	3440 (41.55%)	1942 (40.90%)	1498 (42.42%)	
No	4839 (58.45%)	2806 (59.10%)	2033 (57.58%)	
WCS (h)				.399
<0	1318 (15.92%)	742 (15.63%)	576 (16.31%)	
≥0	6961 (84.08%)	4006 (84.37%)	2955 (83.69%)	

OSA = obstructive sleep apnea, PIR = poverty-income ratio, WCS = weekend catch-up sleep.

### 3.2. Association of WCS with obesity

Table 2 demonstrates the association between WCS and obesity across 3 progressively adjusted logistic regression models. In the unadjusted model, each additional hour of WCS was significantly associated with 3% higher odds of obesity (OR = 1.03, 95% CI: 1.01–1.06; *P* = .0131). This association remained consistent in Model I, which adjusted for age, sex, and race/ethnicity (OR = 1.03, 95% CI: 1.00–1.05; *P* = .0759), and in the fully adjusted Model II (OR = 1.03, 95% CI: 1.00–1.06; *P* = .0872), which additionally accounted for socioeconomic factors (education level, marital status, and PIR), behavioral factors (smoking and total energy intake), clinical comorbidities (hypertension and diabetes), and physical activity, OSA, depression, shift work, and HEI.

**Table 2:** Association between weekend catch-up sleep and obesity.

Exposure	Non-adjusted model	Model I	Model II
WCS	1.03 (1.01–1.06), .0131	1.03 (1.00–1.05), .0759	1.03 (1.00–1.06), .0872
*P* for trend	.8775	.4183	.2620

Model 1 adjust for: sex, age, race.

Model 2 adjust for: sex, age, race, education level, marital status, PIR, hypertension, diabetes, smoking, total energy intake, physical activity, OSA, depression, shift work, and HEI.

OSA = obstructive sleep apnea, PIR = poverty-income ratio, WCS = weekend catch-up sleep.

### 3.3. Restricted cubic spline regression analysis between WCS with obesity

**Figure 2.**
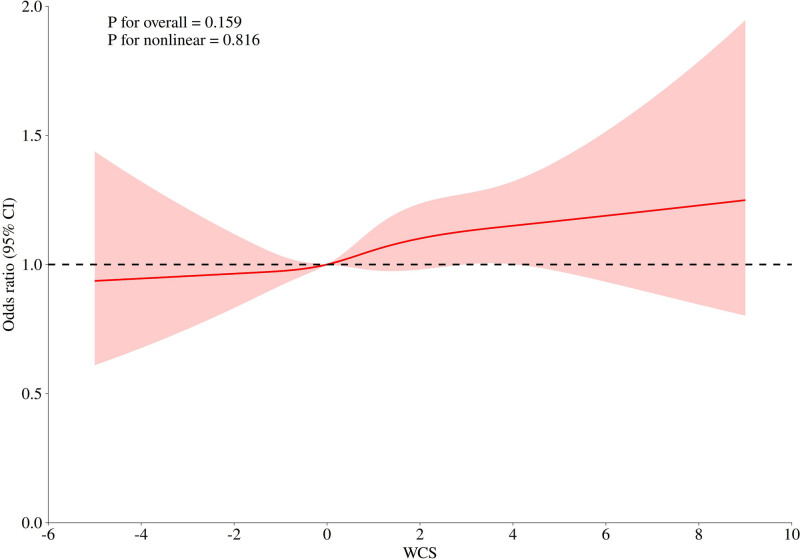
The smoothed curve-fit plot of the dose–response relationship between WCS and obesity. CI = confidence interval, WCS = weekend catch-up sleep.

Figure 2 presents the dose–response association between WCS and the odds of obesity using a restricted cubic spline model. The overall association was not statistically significant (*P* for overall = .159), and the test for nonlinearity also yielded nonsignificant results (*P* = .816). The curve appeared relatively flat when WCS values were negative, with a slight upward trend observed at values above 0. However, given the lack of statistical support, these visual patterns should be interpreted with caution and regarded as exploratory rather than confirmatory.

Table 3 exploratory two-piecewise regression suggested a possible inflection at WCS = 0 hours, with stronger associations for WCS > 0. However, the log-likelihood ratio test (*P* = .113) indicated that the two-piecewise model did not significantly outperform the linear model, and the test for nonlinearity in the spline model was not significant (*P* = .816). Taken together, these findings indicate that the linear model provides the most parsimonious explanation of the association between WCS and obesity.

**Table 3:** Nonlinearity addressing of weekend catch-up sleep (WSC) and obesity.

Obesity	OR (95% CI), *P*-value
WCS	
Fitting model by standard logistic regression	0.14 (0.04 to 0.23), .0058
Fitting model by two-piecewise logistic regression	
Inflection point	0
<0	−0.06 (−0.32 to 0.20), .6559
>0	0.20 (0.07 to 0.32), .0016
*P* for log likely ratio test	.113

Adjusted for all covariates presented in Table 2.

CI = confidence interval, OR = odds ratio, WCS = weekend catch-up sleep.

### 3.4. Subgroup analyses

**Figure 3.**
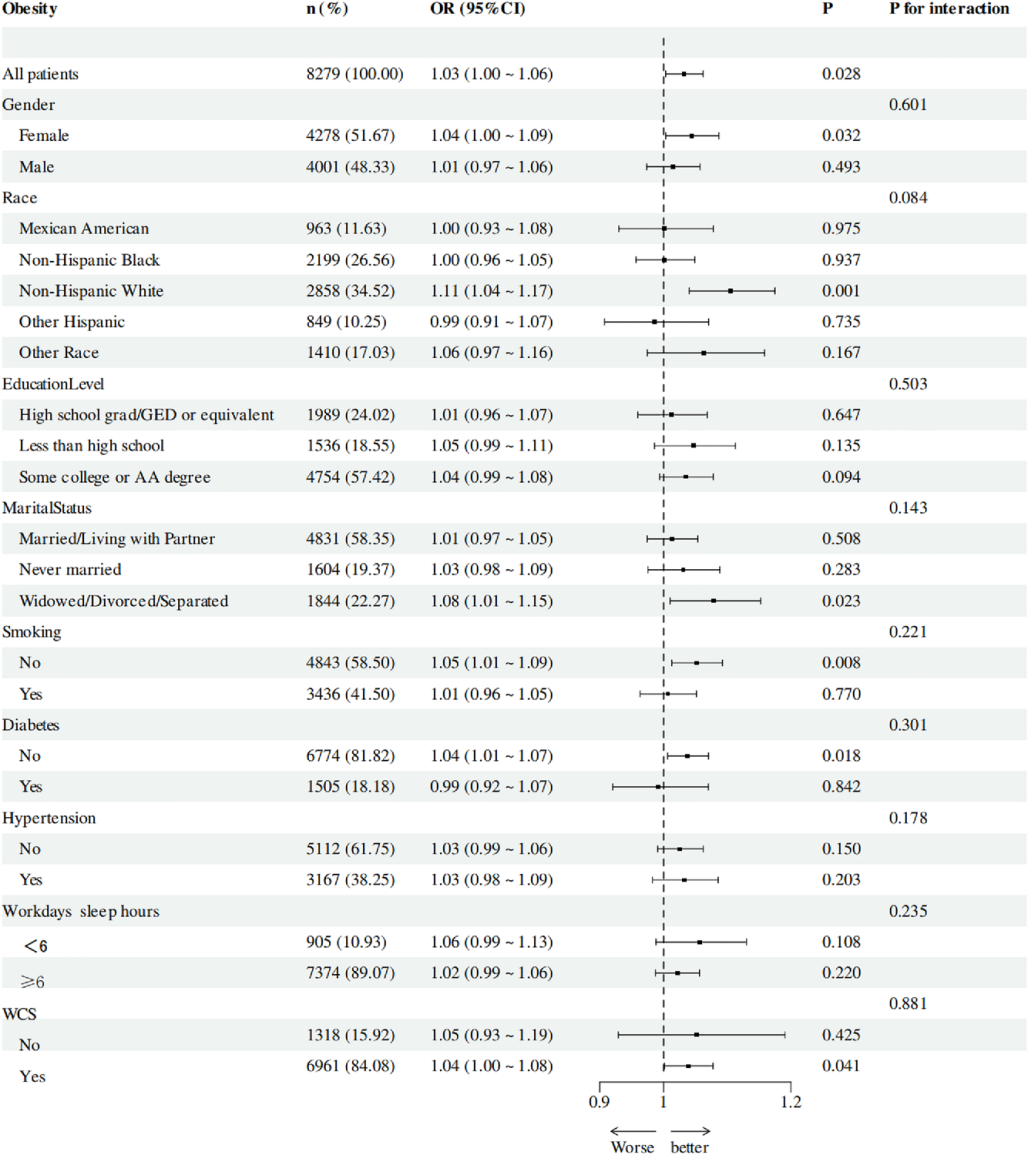
Subgroup analysis for the association between WCS and obesity. CI = confidence interval, OR = odds ratio, WCS = weekend catch-up sleep.

Figure 3 presents subgroup analyses, showing variability in the association between WCS and obesity across population strata. In the overall population, a modest association was observed (OR = 1.03, 95% CI: 1.00–1.06; *P* = .028). By sex, an association was noted among females (OR = 1.04, 95% CI: 1.00–1.09; *P* = .032) but not among males (OR = 1.01, 95% CI: 0.97–1.06; *P* = .493), although no significant interaction was detected (*P* for interaction = .601). Stratified by race/ethnicity, the association appeared stronger among non-Hispanic Black individuals (OR = 1.11, 95% CI: 1.04–1.17; *P* = .001), though the interaction test was not significant (*P* = .084). Across education levels, no statistically significant associations emerged (*P* for interaction = .503), although point estimates suggested a possible trend in participants with less than high school education (OR = 1.05, 95% CI: 0.99–1.11). Marital status-stratified analyses showed a nominal association among widowed, divorced, or separated individuals (OR = 1.08, 95% CI: 1.01–1.15; *P* = .023), yet no interaction effect was observed (*P* = .143). Among nonsmokers, an association was observed (OR = 1.05, 95% CI: 1.01–1.09; *P* = .008), whereas no clear association was seen among smokers (OR = 1.01, 95% CI: 0.96–1.05; *P* = .770), without evidence of interaction (*P* = .221). Similarly, WCS was associated with obesity among nondiabetic individuals (OR = 1.04, 95% CI: 1.01–1.07; *P* = .018), but not among those with diabetes (OR = 0.99, 95% CI: 0.92–1.07; *P* = .842), with no interaction effect (*P* = .301). No effect modification was detected by hypertension status (*P* for interaction = .178), with nonsignificant associations in both normotensive (*P* = .150) and hypertensive (*P* = .203) individuals. When stratified by weekday sleep duration, the association appeared stronger among participants sleeping ≥ 6 hours (OR = 1.04, 95% CI: 1.00–1.08; *P* = .041) than among short sleepers (<6 hours; OR = 1.05, 95% CI: 0.93–1.19; *P* = .425), although the interaction was not significant (*P* = .235). Finally, WCS polarity showed a significant interaction (*P* for interaction = .041), with an association detected only among participants with nonnegative WCS (OR = 1.04, 95% CI: 1.00–1.08; *P* = .041). These subgroup findings should be interpreted as exploratory and hypothesis-generating, given the absence of correction for multiple testing and the potential for chance findings.

Taken together, the subgroup findings indicate limited evidence of effect modification. While some strata (e.g., females, non-Hispanic Black individuals, nonsmokers) showed statistically significant associations, these results must be interpreted with extreme caution given the lack of correction for multiple comparisons. They should be regarded as hypothesis-generating only, warranting further confirmation in future studies.

## 4. Discussion

In this cross-sectional study using nationally representative data from NHANES 2017–2020, we observed a modest but statistically significant positive association between WCS and obesity in U.S. adults. Specifically, each 1-hour increase in WCS was associated with a 3% higher odds of obesity after adjustment for demographic, socioeconomic, behavioral, and clinical factors. Although exploratory analyses suggested a possible deviation from linearity, neither spline regression (*P* for nonlinearity = .816) nor two-piecewise modeling (likelihood ratio test *P* = .113) provided statistical support for a threshold effect. Therefore, the observed association is best interpreted as linear. Exploratory subgroup analyses suggested that the association may be more pronounced among certain groups (e.g., females, non-Hispanic Black individuals, nonsmokers, and those without diabetes). However, these apparent subgroup effects must be interpreted with extreme caution because no correction for multiple testing was applied. Therefore, they should be considered hypothesis-generating rather than confirmatory.

Several recent studies have investigated the link between WCS and metabolic health, including obesity-related outcomes. A cross-sectional analysis using NHANES data (2017–2020) demonstrated that moderate WCS (approximately 0.7–1.0 hours) was associated with a lower risk of insulin resistance.^[21]^ Another study based on NHANES found WCS to be inversely related to markers of systemic inflammation, particularly among individuals with short weekday sleep.^[9]^ Furthermore, a prospective cohort study in Korea reported that adolescents who consistently obtained 1–2 hours of additional weekend sleep had significantly lower BMI over time.^[11]^ These findings mirror our results, in which moderate WCS correlated with reduced obesity odds among U.S. adults, reinforcing the possibility of metabolic compensation through weekend sleep extension.

Conversely, controlled laboratory studies highlight caution. A trial involving cyclical weekday sleep restriction followed by weekend recovery showed no improvement in insulin sensitivity or weight outcomes.^[22]^ In line with these observations, our spline analysis revealed a plateau effect beyond approximately 2 hours of WCS, with no additional reduction in obesity odds at higher levels of catch-up sleep. One possible explanation is that very high WCS reflects irregular sleep timing (a form of “social jetlag”), which can misalign circadian rhythms and potentially offset the metabolic benefits of recovery sleep. Variations in study findings across the literature may be driven by differences in age, cohort characteristics, study settings (observational vs laboratory), and sleep measurement methods. Taken together, these results underscore the complexity of WCS effects: moderate weekend sleep extension was associated with more favorable metabolic indicators in some studies, whereas excessive or erratic catch-up sleep appeared to yield diminishing returns or even counterproductive associations.^[23]^ By leveraging nationally representative U.S. data, our study provides evidence that moderate WCS is associated with lower obesity prevalence, underscoring the importance of considering sleep patterns in obesity research and prevention efforts.

To explore the relationship between WCS and obesity, we employed adjusted logistic regression, subgroup analyses, and trend tests. The inverse association between moderate WCS and obesity risk was more evident among females, non-Hispanic Black individuals, and those without diabetes, suggesting potential metabolic differences across subpopulations. However, this association weakened after adjusting for demographic, health, and lifestyle factors, indicating possible confounding. These findings highlight the complex interplay between sleep and weight and underscore the need for longitudinal studies to determine whether WCS is a beneficial adaptation or a marker of sleep irregularity with varied effects.^[24]^

Several biological pathways may explain the association between WCS and obesity. Sleep restriction alters appetite-regulating hormones by reducing leptin and increasing ghrelin levels, thereby promoting overeating and weight gain. WCS may partially restore this hormonal balance and improve appetite control.^[25]^ Insufficient sleep also impairs insulin sensitivity and elevates fasting glucose, increasing metabolic risk. Recovery sleep may reverse these effects, especially in chronically sleep-deprived individuals.^[26–28]^ Furthermore, disrupted sleep elevates inflammatory markers such as C-reactive protein and interleukin 6, which are linked to obesity pathogenesis. WCS may attenuate this low-grade inflammation.^[9,29]^ However, excessive or inconsistent WCS might reflect irregular sleep timing (i.e., social jetlag) that misaligns circadian rhythms and undermines metabolic regulation. Notably, our study did not measure circadian phase or sleep timing directly, so this interpretation is speculative.^[30,31]^ Neuroimaging studies also show that sleep loss enhances brain reward responses to high-calorie foods, and regular recovery sleep may help normalize this effect.^[32,33]^ Overall, the interplay of hormonal, inflammatory, circadian, and neural factors suggests that moderate WCS could have metabolic benefits, but its effects are context-dependent and require further investigation.

Importantly, the effect size observed in our primary analysis (OR = 1.03 per additional hour of WCS) was very modest. Although statistically significant in this large sample, such a small magnitude of association is unlikely to be clinically meaningful at the individual level. For example, a 3% increase in odds of obesity per hour of catch-up sleep would not, in isolation, justify clinical recommendations to alter sleep behavior. However, given the high prevalence of obesity and widespread practice of WCS in the population, even small associations may accumulate to yield measurable effects at the population level. Thus, our findings should be interpreted as hypothesis-generating and warrant further confirmation in longitudinal and mechanistic studies.

These observations may be especially pertinent for metabolically vulnerable subgroups (e.g., individuals with hypertension, diabetes, or highly irregular schedules), for whom even modest sleep pattern disruptions could have outsized metabolic effects.^[34]^ Although our findings suggest a possible beneficial association of moderate WCS with lower obesity odds, the cross-sectional design precludes any causal inference. Additionally, the observed relationship could partly reflect reverse causation: individuals with obesity may engage in more catch-up sleep due to greater sleep needs. Obesity is often accompanied by OSA, chronic inflammation, and other metabolic disturbances that increase fatigue and sleep drive, potentially leading to increased weekend sleep duration in those with higher BMI. This scenario would mean that obesity leads to more WCS (rather than WCS preventing obesity), highlighting the importance of cautious interpretation. To better understand this relationship, future research should employ longitudinal designs with objective sleep assessments and metabolic biomarkers. Moreover, consideration of sleep quality, chronotype, and behavioral factors could help elucidate interindividual differences in the health impact of WCS.

### 4.1. Limitations

This study has several limitations that warrant consideration. Firstly, due to its cross-sectional nature, the analysis cannot establish a definitive causal relationship between WCS and obesity. Secondly, reliance on self-reported sleep duration introduces the potential for recall inaccuracies and misclassification, which may affect the reliability of the exposure assessment. Third, NHANES lacks objective sleep measures and data on sleep quality or circadian phase markers. Fourth, although we adjusted for a wide range of potential confounders – including physical activity, diet quality (HEI), OSA, depression, and shift work – the influence of residual or unmeasured confounding cannot be completely excluded. For instance, factors such as psychosocial stress, environmental exposures, or other comorbidities not captured in NHANES may still influence the observed associations. Lastly, our operational definition of WCS may not fully capture individual variability in sleep behaviors, chronotype, or circadian alignment. We lacked data on sleep timing or circadian phase, so WCS cannot be interpreted as a precise measure of circadian misalignment.

## 5. Conclusion

In this cross-sectional analysis of U.S. adults from NHANES 2017–2020, WCS showed a modest linear association with obesity. Although exploratory plots hinted at a potential threshold around 0 hours, formal model comparisons did not support nonlinearity; thus, a linear specification is the most parsimonious and statistically supported. Subgroup findings – suggesting stronger associations among women and non-Hispanic Black participants – should be interpreted cautiously given multiple testing and lack of prespecification, and are best viewed as hypothesis-generating. The small effect size limits immediate clinical or public health relevance. Moreover, without objective measures of sleep timing, we cannot determine the role of circadian misalignment. Prospective studies incorporating objective sleep metrics are needed to clarify causality and underlying biological pathways.

## Acknowledgments

We sincerely thank the National Center for Health Statistics, part of the Centers for Disease Control and Prevention, for their dedicated efforts in developing and maintaining the NHANES database. Their contributions to data collection, management, and public accessibility have been invaluable to advancing public health research.

## Author contributions

**Funding acquisition:** Dongxiao Huang.

**Investigation:** Minmin Zhu.

**Methodology:** Minmin Zhu.

**Visualization:** Jinfang Zeng.

**Writing – original draft:** Jinfang Zeng, Jinjin Jian.

**Writing – review & editing:** Jinjin Jian.
